# Hesitancy, Trust and Individualism in Vaccination Decision-Making

**DOI:** 10.1371/currents.outbreaks.49dba84ad4146de33706b1f131d7caa3

**Published:** 2015-02-25

**Authors:** Jonathan E Suk, Pierluigi Lopalco, Lucia Pastore Celentano

**Affiliations:** European Centre for Disease Prevention and Control, Stockholm, Sweden; European Centre for Disease Prevention and Control, Stockholm, Sweden; European Centre for Disease Prevention and Control, Stockholm, Sweden

**Keywords:** vaccine hesitancy

## Related Articles

The article is part of the *PLOS Currents Outbreaks *"Vaccine Hesitancy Collection".

## Editorial

Based on recent trends, outbreaks of measles and other vaccine-preventable diseases could be more commonplace in the coming years, even in countries where such diseases have been considered eliminated or under control. In 2014, the United States reported over 600 cases of measles, far and away the highest number over the past decade.^1^ In the European Union, where measles is still endemic, this figure is an order of magnitude higher, with 3840 reported cases in the rolling twelve month period between December 2013 and November 2014.^2^ Measles continues to be challenge in many additional parts of the world, with countries such as Canada, Brazil, Vietnam and China all reporting recent increases in measles incidence and/or current outbreaks.^3^

The willingness or reticence of individuals to vaccinate themselves and their children can have profound impacts not only for their own health and wellbeing, but for herd immunity and public health more widely. As noted in Europe for measles, each percentage point increase in national vaccination coverage contributes to a significant reduction in the overall burden of disease.^4^ Thus, when contemplating immunisations, individuals may be assessing personal risks and benefits – but they are impacting societal ones.

Very recently, a measles outbreak at a prominent Californian theme park sparked wide-scale public debate in the United States, ultimately reaching the highest political circles, with President Obama affirming on national television that “the science is pretty indisputable.”^5^ Other US politicians, meanwhile, situated the vaccination debate in the context of broader political discourses, such as the right to individual freedom versus state intervention.^6^ The latter is indeed an important factor contributing to lower than ideal vaccination coverage amongst some groups, but several other factors are known to create barriers to vaccination.^7^ These can include complacency and neglect; the desire for “toxin-free” lifestyles; varying religious beliefs; public interpretations of risk and benefits of vaccines that are at odds with medical consensus; and, somewhat relatedly, a lack of trust in scientific and medical establishments.

If the recent Californian measles outbreak (and the reaction to it) is instructive of anything, it is perhaps simply that vaccine hesitancy and other barriers to vaccination (e.g. among hard-to-reach populations) is an issue that appears to be increasingly pressing and politicized in many parts of the world. It therefore warrants much greater attention from public health and epidemiology, medical sociology, anthropology, and the behavioural, economic and political sciences. Recognizing this need, *PLOS Currents: Outbreaks* and the *European Centre for Disease Prevention and Control (ECDC)* issued a call for papers aimed at building upon the insights collected from a 2013 workshop on the topic of vaccine hesitancy.^8^^,^ 9

The papers presented in this collection offer a unique and important contribution to the field. Peretti-Watel *et al.* 10 and Larson *et al.* 11 stress the importance of clarifying the language around vaccine hesitancy and confidence. The former notes the consistencies and inconsistencies of the ways in which the term has been used, offering much needed clarity in this emerging domain of research. They convincingly argue that is helpful to view vaccine hesitancy as a decision-making process. Recognising it as such requires attention to the many factors that may affect it. As addressed in this collection of papers, these can include the important but often overlooked role of social discourses (Abeysinghe^12^); age and social position, as discussed in the context of measles vaccination coverage in Germany (Schuster *et al.* 13); and perceptions of the severity of disease, noted in a study of the intentions of US women to receive antenatal influenza and Tdap vaccines (Chamberlain *et al.* 14).

The theme of trust and of vaccine confidence, meanwhile, resonates across each of the papers in this issue. As Peretti-Watel *et al.* 10 note, the parallels between vaccine hesitancy and the sociological theorisation on risk developed over twenty years ago are striking. For example, a particularly salient concept of risk society theory for vaccine hesitancy is reflexive modernisation, a process through which the risks produced by science and technology attract both attention and scepticism. This is accompanied by a growing lack of public trust in governments and scientific institutions, leading individuals to “privatize” their risk management decisions.^15^^,^ 16 Such a dynamic certainly appears to be at play when considering vaccination. As some recent studies have demonstrated, there is a connection between trust to broader social structures and individuals’ decisions to vaccinate in both Europe and the United States.^17^^,^ 18

One of the critiques of risk society theory has been the argument that it is not particularly relevant beyond the “West”. Irrespective of whether or not this is the case, vaccine hesitancy certainly is. Larson *et al.* 11 present findings belonging to a global vaccine confidence survey. Data from Georgia, India, Pakistan, the UK and Nigeria indicate that for each of these countries, confidence in immunisation is linked to confidence in health systems more generally. Although vaccine hesitancy is relatively rare – and vaccine refusals even rarer – even small groups can undermine the success of immunisation programmes. This, they note, begs the question, “How much confidence is enough?” It is one of many pressing questions that the papers in this issue begin to address – and one that will require much further research in the coming years.

## Competing Interests

The authors have declared that no competing interests exist.
